# High Transmissibility During Early HIV Infection Among Men Who Have Sex With Men—San Francisco, California

**DOI:** 10.1093/infdis/jiu831

**Published:** 2014-12-26

**Authors:** T. Déirdre Hollingsworth, Christopher D. Pilcher, Frederick M. Hecht, Steven G. Deeks, Christophe Fraser

**Affiliations:** 1 Warwick Infectious Disease Epidemiology Research, Warwick Mathematics Institute; 2 School of Life Sciences, University of Warwick, Coventry; 3 Department of Clinical Sciences, Liverpool School of Tropical Medicine; 4 MRC Centre for Outbreak Analysis and Modelling, Department for Infectious Disease Epidemiology, School of Public Health, Imperial College London, United Kingdom; 5 HIV/AIDS Division, Department of Medicine, San Francisco GeneralHospital, University ofCalifornia–San Francisco

**Keywords:** HIV, transmission rate, early infection, men who have sex with men, San Francisco

## Abstract

We estimate the relative transmission rate in early versus later infection among men who have sex with men (MSM) in San Francisco, California, by studying the characteristics of a sample of transmitters, recruited through newly diagnosed, recently infected MSM between 1996 and 2009. Of 36 transmitters identified, 9 were determined on the basis of testing history and serologic testing to have been recently infected. The unadjusted odds ratio of transmitting during early infection was 15.2 (95% confidence interval [CI], 6.3–33.4; *P* < .001); the odds ratio was 8.9 (95% CI, 4.1–19.4) after adjustment for self-reported antiretroviral treatment. This high transmissibility could be due to both high infectiousness and high rates of sex partner change or concurrent partnerships.

Estimates of the relative transmission rate of human immunodeficiency virus (HIV) during early infection are essential for understanding the dynamics of transmission and the likely impact of intervention programs [1]. Early infection is associated with high transmission rates among heterosexuals, but there are limited estimates among men who have sex with men (MSM) [2]. High infectivity in early infection is thought to be due to a combination of high viral loads, viral phenotype, or host risk factors, such as coinfection. Studies that stratify transmission rates among MSM by viral load show a strong association between high viral loads and transmission rates, suggesting that early infection, characterized by high viral loads in semen [3], is highly infectious among MSM. A recent review of HIV transmission by anal intercourse found no significant difference in transmission probability per unprotected penile-anal act among the heterosexual population, compared with the MSM population [4], and early infection has been shown to be highly infectious among heterosexuals [5, 6].

Phylogenetic studies of MSM-dominated epidemics have shown a high degree of clustering among samples from early infection, suggesting that chains of transmission during early infection are contributing to a large proportion of transmissions (from 10% to 50% [7]), a consequence of both differential infectiousness, the stage of the epidemic, and the sexual risk behavior in the population [8]. Direct estimation of the relative infectiousness of early infection in this way is inherently challenging but can be done well [9]. An alternative approach is to use viral phylogenies to identify likely transmission pairs within a longitudinally studied cohort, using clinical data to infer the direction of transmission, and to compare the pairs to randomly chosen members of the cohort to quantify the risk factors associated with transmission; Fisher et al estimated a rate ratio of transmission during early infection of 3.88 (95% confidence interval [CI], 1.76–8.55; *P* = .0008) [10]. As with all indirect methods, including the method used in the analysis described below, these estimates are affected by the dynamics of each particular epidemic, including variable behavior, diagnosis, and treatment patterns. Since all of these estimates each have their own limitations, multiple estimates of transmission rates using different methods are needed to develop a better understanding of the dynamics of the HIV epidemic.

We estimated the relative transmission rate of early infection among MSM in San Francisco, California, by comparing the characteristics of a sample of transmitters with those of the general HIV-infected population. This is similar to a case-control study in which cases are source partners known to have transmitted HIV to their partners and controls are the HIV-infected population. We assume that the source partners are a random sample of transmitters, ie, that the characteristics of both recent and chronic transmitters are similar to the characteristic of individuals with this stage of infection in the larger population. This is similar to the method used by Fisher et al [10] but differs principally by the methods used to identify transmitters and to stage early infection.

## METHODS

The relative infectiousness of recently and chronically infected individuals can be estimated by comparing the distribution of recently infected individuals in a sample of transmitters with that in the estimated HIV-infected MSM population at the time of recruitment. A group of HIV-infected MSM were identified by recruitment of their newly infected sex partners and genetic confirmation of transmission (Supplementary Materials) [11]. This was a secondary analysis of anonymized data.

The present analysis makes an assumption that the method of selection gives an unbiased sample of transmitters because they were all recruited in a similar way. For this reason, we included only transmitters who were initially identified as being a potential source partner for a new recently infected index case.

To estimate the role of early infection in transmission, the stage of infection for the majority of transmitters was characterized using an enzyme immunoassay (EIA) (Supplementary Materials).

To allow for reduced transmission during chronic infection due to treatment, which could inflate the estimate of the relative transmissibility during early infection, treatment status was also requested from each transmitter.

The comparison group for this analysis was the HIV-infected MSM population in San Francisco. The San Francisco Department of Public Health publishes yearly estimates of the HIV-infected population, including those with undiagnosed infection and those with incident infection (Supplementary Materials). Since newly infected individuals were only in the early phase of infection for a portion of the year, we approximated their presence in the epidemic by using the duration of the mean interval between the time at which an HIV-infected individual tests positive for HIV antibody by a sensitive EIA and the time at which the individual tests negative for HIV antibody by a less sensitive EIA, with a cutoff of approximately 164 days [12]. If *k* is defined as the number of new infections in a given year, approximately 164/365*k person-years of potential transmission during early infection were assumed to have occurred.

The distribution of transmitters and HIV-infected individuals can be compared to estimate the odds ratio (OR) of transmission during early infection; that is, the relative transmission rate in early infection, which is a combination of biological and behavioral factors. These ORs were obtained using standard methods, implemented using the function epi.2by2 in the package epiR for R, including the Breslow Day test for homogeneity across years [13].

## RESULTS

From 1997 to 2009, 656 index cases with early infection were seen, and partners were identified in 69 (10.5%; from 2000 to 2003, when the most partners were recruited, transmitters were successfully identified for 43 of 209 index cases [21%]). Twenty-seven transmitting pairs were excluded because (1) the index case was found not to be recently infected on further analysis (6 pairs), (2) the transmitter was already in the cohort (11 pairs), (3) staging information was unavailable for the source partner (9 pairs), or (4) a third individual was possibly the source for both partners (1 pair). This left 42 transmitting pairs, of whom 36 were MSM. Of these, 9 had individuals who were in the early phase of infection, and the remaining 27 had individuals who were in the chronic phase of infection (Table 1). The mean duration of infection for these 9 recently infected transmitters was estimated to be 70 days (median duration, 72 days; range, 24–153 days). Of the 27 chronic transmitters studied here, 6 were reported as receiving or probably receiving antiretroviral treatment (ART) for HIV, although most if not all of these treated sources lacked evidence of having achieved and maintained suppression of virus to levels below the limits of detection. Newly infected individuals made up approximately 5% of the HIV-infected population in the whole year (scaling to account for the proportion of the year in early infection, this is approximately 2%) but 25% of transmitters (Table 1), suggesting that recently infected individuals are highly overrepresented in transmitters and contribute a considerable proportion of onward transmission. Conversely, treated chronically infected individuals are underrepresented in the transmitters (17%), compared with the HIV-infected population (52%; Table 1).

**Table 1.** Characteristics of Human Immunodeficiency Virus (HIV) Transmitters and the HIV-Infected Population in San Francisco

Year	HIV Transmitters				HIV-Infected Population			
	Early	Chronic			Early	Chronic		
		Untreated	Treated	Total No.	(Whole Year)	Untreated	Treated	Total No.
1997	0 (0)	2 (100)	0 (0)	2	283 (2)	7071 (59)	4628 (39)	11 983
1998	1 (50)	1 (50)	0 (0)	2	399 (3)	6385 (52)	5586 (45)	12 370
2000	2 (22)	6 (67)	1 (11)	9	631 (5)	6142 (47)	6371 (48)	13 146
2001	2 (22)	6 (67)	1 (11)	9	748 (6)	5984 (44)	6801 (50)	13 534
2002	1 (17)	2 (33)	3 (50)	6	752 (5)	5876 (43)	7193 (52)	13 822
2003	1 (33)	2 (67)	0 (0)	3	757 (5)	5768 (41)	7585 (54)	14 111
2004	0 (0)	1 (100)	0 (0)	1	762 (5)	5819 (40)	7817 (54)	14 399
2008	2 (67)	1 (33)	0 (0)	3	697 (5)	5079 (35)	8869 (61)	14 646
2009	0 (0)	0 (0)	1 (100)	1	659 (5)	4689 (32)	9131 (63)	14 480
Overall	9 (25)	21 (58)	6 (17)	36	5688 (5)	52 813 (43)	63 981 (52)	122 491

Data are no. (%) of individuals, unless otherwise indicated. Additional information is available in the Supplementary Materials.

Early infection was estimated to be a highly infectious period (crude OR [vs chronic infection], 15.2; 95% CI, 6.3–33.4; *P* < .001), with evidence that the transmissibility was not the same across years (*P* = .69). The analysis also suggested a marked reduction in the odds of transmitting due to treatment (OR [vs no treatment], 0.25; 95% CI, .08–.63; *P* = .001). After adjustment for the impact of treatment, the OR for early infection decreased to 8.9 (95% CI, 4.1–19.4; *P* < .001).

To investigate the impact of the uncertainty in the duration of early infection, the estimates were repeated for the 95% CI on the estimated duration of early infection in the assay [12]; for 139 days, the adjusted OR was 10.4 (95% CI, 4.8–22.8), and for 199 days, the adjusted OR was 7.3 (95% CI, 3.3–15.9). Sensitivity to overestimation of the number of early infections was considered by rerunning the analysis at a cutoff of 0.75 for the early infection assay and a consequent duration of 129 days [12], giving an additional 2 cases during chronic infection and an adjusted OR of 7.0 (95% CI, 3.4–18.7).

## DISCUSSION

When an event is rare, the OR is a good approximation of the relative risk. Therefore, an OR can be compared with previous estimates of the relative transmission rate of early infection. An estimated 4–19-fold greater adjusted odds of transmission during early infection, compared with chronic infection, lies at the middle to higher end of estimates for both MSM and heterosexual transmission [4, 14] but overlaps estimates for heterosexual partners in general [8] and for heterosexual partners who engage in anal sex [4]. The estimated reduction in transmission is based on self-reported ART use without confirmation of viral suppression and is thus unsurprisingly lower than that associated with validated ART use with confirmed viral suppression [15]. It should be remembered that the estimate of transmissibility during early infection is a combination of both behavior and biology, the individual effects of which cannot be disentangled with this study design.

The role of high infectivity in population-level transmission is limited by the short duration of this stage of infection but will be increased by high rates of partner change [8]. Because of inevitable delays in identifying transmission and transmitters, our definition of early infection at the time of transmission is defined by the stage of infection of the transmitter at the time of identification some months later. We therefore cannot be certain of the exact stage of infection at the time of transmission, but this increased risk is an estimate of infectiousness during the first 6–9 months of infection. Given this interval, these are very high estimates. A worrying consequence of high transmissibility for MSM during early infection is that it would speed the epidemic growth rate in expanding or re-expanding MSM epidemics. Thus, this study highlights the need for sustained HIV-prevention efforts to help reduce HIV transmission among MSM and for finding ways to more effectively diagnose infection and treat MSM with early HIV infection to prevent reversal of hard-won gains.

The main limitations of our study are the assumptions that the source population in this study represents all sources in San Francisco and that our data are an unbiased sample of transmitters. Short of performing a prospective study of all potential transmitters and their partners, the study design used is the only one we are aware of to address these questions. Although we are not aware of any systematic bias in our assumption, it is possible that the networks dominated by transmisssion in early infection in San Francisco are more integrated and, hence, more likely to present to a research setting as identified partners. Also, during the period of observation, a substantial proportion of the chronically infected population in San Francisco was receiving effective ART. This highlights the need for further studies of this type.

## Supplementary Material

Supplementary Data
