# Supplementary material for: High Transmissibility During Early HIV Infection Among Men Who Have Sex With Men—San Francisco, California
Source: J Infect Dis. 2014 Dec 26;211(11):1757–60. doi: 10.1093/infdis/jiu831 (PMC4425938; doi:10.1093/infdis/jiu831)
Supplement: Supplementary Data [file supp_211_11_1757__index.html]

High infectivity of early HIV infection in men who have sex with men in San Francisco — High Transmissibility During Early HIV Infection Among Men Who Have Sex With Men—San Francisco, California — High Transmissibility During Early HIV Infection Among Men Who Have Sex With Men—San Francisco, California — Supplementary Data 

# High Transmissibility During Early HIV Infection Among Men Who Have Sex With Men—San Francisco, California

## Supplementary Data

Supplementary Data

**Files in this Data Supplement:**

- Supplementary Data - Pdf file
- Supplementary Figure 1 - pdf file
