# Supplementary material for: High Transmissibility During Early HIV Infection Among Men Who Have Sex With Men—San Francisco, California
Source: J Infect Dis. 2014 Dec 26;211(11):1757–60. doi: 10.1093/infdis/jiu831 (PMC4425938; doi:10.1093/infdis/jiu831)
Supplement: Supplementary Data [file supp_jiu831_jiu831supp.pdf]

High infectivity of early HIV infection in men who have sex with men in San Francisco

T. Déirdre Hollingsworth[1,2,3], Christopher D. Pilcher*[4], Frederick M. Hecht[4], Steven G. Deeks[4], Christophe Fraser*[5]

[1]Warwick Mathematics Institute, University of Warwick, Coventry, CV4 7AL, UK; [2]School of Life Sciences, University of Warwick, Coventry, CV4 7AL, UK; [3]Department of Clinical Sciences, Liverpool School of Tropical Medicine, Liverpool, UK; [4]HIV/AIDS Division, Department of Medicine, San Francisco General Hospital, University of California, USA; [5]MRC Centre for Outbreak Analysis and Modelling, Department for Infectious Disease Epidemiology, School of Public Health, Imperial College London, UK

*contributed equally

Supplementary text

This supplementary text gives additional information on the data sources, firstly the cohort of transmitters and secondly the HIV infected population in San Francisco.

1. Cohort of transmitters

In order to identify MSM who had recently transmitted HIV in San Francisco, we recruited sexual partners of participants in the UCSF Options study of acute and recently HIV-infected MSM in San Francisco [32]. Acute or recent HIV infection subjects in the Options cohort were enrolled based on evidence of ongoing HIV sero-conversion – e.g. having a negative HIV test within 6 months, or a clinical and exposure history suggesting recent infection, confirmed a very low result on a detuned antibody assay consistent with infection of less than six months duration. These criteria were distinct from the methodology used to identify stage of infection in the likely transmitter - because the "transmitters" were typically tested several months after the transmission event, the definition of recent infection (at transmission to the initially identified partner), included transmitters who were infected up to a year (at the time of inclusion in our study). Further details below.

All acute or recently infected index cases were interviewed with regard to their potential source partners, and attempts were made to bring partners in. When index case-partner transmission pairs were confirmed by HIV testing of the partner, viral genetics were compared for confirmation of a suspected transmission relationship. We then assessed which partner was the likely transmitter based on review of detailed sexual histories, clinical history and all available HIV testing results (including less-sensitive EIA testing) [32]. This was a secondary analysis of anonymized data. Written informed consent was obtained from all potential source and recipient partners using protocols that were reviewed and approved by the UCSF Institutional Review Board.

The present analysis makes an assumption that the method of selection gives an unbiased sample of transmitters, because they were all recruited in a similar way. For this reason, we included only transmitters who were initially identified as being a potential source partner for a new recently infected index case; index cases who were found to be the transmitter in the partnership.

In order to estimate the role of early infection in transmission, the stage of infection for each transmitter was characterized using a less-sensitive HIV antibody immunoassay designed to discriminate

recent HIV infection. For this study, the majority of transmitters (33 out of 36) were characterized using an enzyme immunoassay (LS-EIA) Vironostika-based HIV-1 antibody test. The remaining 3 transmitters, who were studied after the Vironostika kit was discontinued, were tested using a less-sensitive adaptation of the Ortho Vitros ECi HIV-1 antibody EIA (LS-Vitros)[35]. In order to classify transmitters as recent vs. non-recent infection for this study, cut-offs were selected that corresponded to an average infection time of one year post-seroconversion. Cut-offs of 1.0 standardized optical density (OD) for the Vironostika LS-EIA [33, 34], and 20 for the LS-Vitros assay [35] were used. 8 of 33 were classified as recent HIV infection by Vironostika LS-EIA and 1 of 3 were classified as recent by the LS-Vitros using these criteria.

2. San Francisco HIV infected population

Monitoring data: The San Francisco Department of Public Health publishes extensive reports on HIV in the city in an annual report [36]. This includes data on the number of cases diagnosed and living with HIV/AIDS stratified by risk group, together with an estimate of those on treatment, in an annual report (Figure A1). Prior to 2003 the annual reports included only AIDS cases (Figure A1a grey lines), but from 2003 onwards the reports additionally include HIV non-AIDS cases (Figure A1a black lines), having previously reported AIDS cases only (Figure A1a, grey lines). Every four to five years the San Francisco Department of Public Health publish consensus estimates of the population at risk of HIV, the number of people living with HIV/AIDS and the number of new infections in the previous year (Figure A1 blue lines).

Estimates used for HIV infected and new infections: Since we require estimates of the whole HIV infected population in our analysis, we linearly extrapolate between the consensus estimates for the total HIV infected population and the number of new infections in that year to obtain the numbers used in our analysis (Figure A1b blue crosses).

Percentage on treatment: Since treatment can only follow diagnosis, the percentages of cases on ART (Figure A1a, open symbols) is a good estimate of the number on treatment for the whole population. Due to the change in reporting in 2003, we extrapolate from the data for HIV and HIV/AIDS cases backwards in time using the data from AIDS cases pre-2003 (Figure A1a, grey crosses). It should be noted that Percent of ART is a crude, lower level estimate calculated among persons living with HIV/AIDS who are known to have started ART by the end of the year. ART data are incomplete for persons who were diagnosed or started ART in recent years and whose ART information has not been updated, those whose medical records are not available for review, or those who moved out of San Francisco.

References

1. Cohen MS, Dye C, Fraser C, Miller WC, Powers KA, Williams BG. HIV treatment as prevention: debate and commentary--will early infection compromise treatment-as-prevention strategies? PLoS Med **2012**; 9:e1001232.
2. Wawer MJ, Gray RH, Sewankambo NK, et al. Rates of HIV-1 transmission per coital act, by stage of HIV-1 infection, in Rakai, Uganda. J Infect Dis **2005**; 191:1403-9.
3. Hollingsworth TD, Anderson RM, Fraser C. HIV-1 transmission, by stage of infection. J Infect Dis **2008**; 198:687-93.
4. Pinkerton SD. Probability of HIV Transmission During Acute Infection in Rakai, Uganda. AIDS and behavior **2008**; 12:677-84.
5. Boily MC, Baggaley RF, Wang L, et al. Heterosexual risk of HIV-1 infection per sexual act: systematic review and meta-analysis of observational studies. Lancet Infect Dis **2009**; 9:118-29.
6. Powers KA, Poole C, Pettifor AE, Cohen MS. Rethinking the heterosexual infectivity of HIV-1: a systematic review and meta-analysis. Lancet Infect Dis **2008**.
7. Baggaley RF, White RG, Boily MC. HIV transmission risk through anal intercourse: systematic review, meta-analysis and implications for HIV prevention. Int J Epidemiol **2010**; 39:1048-63.
8. Baggaley RF, White RG, Boily MC. Infectiousness of HIV-infected homosexual men in the era of highly active antiretroviral therapy. AIDS **2010**; 24:2418-20.
9. Wilson DP, Law MG, Grulich AE, Cooper DA, Kaldor JM. Relation between HIV viral load and infectiousness: a model-based analysis. Lancet **2008**; 372:314-20.
10. Pedraza MA, del Romero J, Roldan F, et al. Heterosexual transmission of HIV-1 is associated with high plasma viral load levels and a positive viral isolation in the infected partner. J Acquir Immune Defic Syndr **1999**; 21:120-5.
11. Quinn TC, Wawer MJ, Sewankambo N, et al. Viral load and heterosexual transmission of human immunodeficiency virus type 1. Rakai Project Study Group. N Engl J Med **2000**; 342:921-9.
12. Fideli US, Allen SA, Musonda R, et al. Virologic and immunologic determinants of heterosexual transmission of human immunodeficiency virus type 1 in Africa. AIDS Res Hum Retroviruses **2001**; 17:901-10.
13. Tovanabutra S, Robison V, Wongtrakul J, et al. Male viral load and heterosexual transmission of HIV-1 subtype E in northern Thailand. J Acquir Immune Defic Syndr **2002**; 29:275-83.
14. Attia S, Egger M, Muller M, Zwahlen M, Low N. Sexual transmission of HIV according to viral load and antiretroviral therapy: systematic review and meta-analysis. AIDS **2009**; 23:1397-404.
15. Pilcher CD, Tien HC, Eron JJ, Jr., et al. Brief but efficient: acute HIV infection and the sexual transmission of HIV. J Infect Dis **2004**; 189:1785-92.
16. Cohen MS, Chen YQ, McCauley M, et al. Prevention of HIV-1 infection with early antiretroviral therapy. N Engl J Med **2011**; 365:493-505.
17. Celum C, Wald A, Lingappa JR, et al. Acyclovir and transmission of HIV-1 from persons infected with HIV-1 and HSV-2. N Engl J Med **2010**; 362:427-39.
18. Donnell D, Baeten JM, Kiarie J, et al. Heterosexual HIV-1 transmission after initiation of antiretroviral therapy: a prospective cohort analysis. Lancet **2010**; 375:2092-8.
19. Leynaert B, Downs AM, de Vincenzi I. Heterosexual transmission of human immunodeficiency virus: variability of infectivity throughout the course of infection. European Study Group on Heterosexual Transmission of HIV. Am J Epidemiol **1998**; 148:88-96.
20. Brenner BG, Roger M, Moisi DD, et al. Transmission networks of drug resistance acquired in primary/early stage HIV infection. AIDS **2008**; 22:2509-15.
21. Pao D, Fisher M, Hue S, et al. Transmission of HIV-1 during primary infection: relationship to sexual risk and sexually transmitted infections. AIDS **2005**; 19:85-90.

22. Yerly S, Vora S, Rizzardi P, et al. Acute HIV infection: impact on the spread of HIV and transmission of drug resistance. AIDS **2001**; 15:2287-92.
23. Bezemer D, van Sighem A, Lukashov VV, et al. Transmission networks of HIV-1 among men having sex with men in the Netherlands. AIDS **2010**; 24:271-82.
24. Cuevas MT, Munoz-Nieto M, Thomson MM, et al. HIV-1 transmission cluster with T215D revertant mutation among newly diagnosed patients from the Basque Country, Spain. J Acquir Immune Defic Syndr **2009**; 51:99-103.
25. Lewis F, Hughes GJ, Rambaut A, Pozniak A, Leigh Brown AJ. Episodic sexual transmission of HIV revealed by molecular phylodynamics. PLoS Med **2008**; 5:e50.
26. Leigh Brown AJ, Lycett SJ, Weinert L, Hughes GJ, Fearnhill E, Dunn DT. Transmission Network Parameters Estimated From HIV Sequences for a Nationwide Epidemic. J Infect Dis **2011**; 204:1463-9.
27. Brenner BG, Roger M, Stephens D, et al. Transmission Clustering Drives the Onward Spread of the HIV Epidemic Among Men Who Have Sex With Men in Quebec. J Infect Dis **2011**; 204:1115-9.
28. Volz EM, Ionides E, Romero-Severson EO, Brandt MG, Mokotoff E, Koopman JS. HIV-1 Transmission during Early Infection in Men Who Have Sex with Men: A Phylodynamic Analysis. PLoS Med **2013**; 10:e1001568.
29. Volz EM, Koopman JS, Ward MJ, Brown AL, Frost SD. Simple epidemiological dynamics explain phylogenetic clustering of HIV from patients with recent infection. PLoS computational biology **2012**; 8:e1002552.
30. Fisher M, Pao D, Brown AE, et al. Determinants of HIV-1 transmission in men who have sex with men: a combined clinical, epidemiological and phylogenetic approach. AIDS **2010**; 24:1739-47.
31. Abu-Raddad LJ, Longini IM, Jr. No HIV stage is dominant in driving the HIV epidemic in sub-Saharan Africa. AIDS **2008**; 22:1055-61.
32. Hecht FM, Hartogensis W, Bragg L, et al. HIV RNA level in early infection is predicted by viral load in the transmission source. AIDS **2010**; 24:941-5.
33. Hecht FM, Busch MP, Rawal B, et al. Use of laboratory tests and clinical symptoms for identification of primary HIV infection. AIDS **2002**; 16:1119-29.
34. Janssen RS, Satten GA, Stramer SL, et al. New testing strategy to detect early HIV-1 infection for use in incidence estimates and for clinical and prevention purposes. JAMA **1998**; 280:42-8.
35. Keating SM, Hanson D, Lebedeva M, et al. Lower-sensitivity and avidity modifications of the vitros anti-HIV 1+2 assay for detection of recent HIV infections and incidence estimation. J Clin Microbiol **2012**; 50:3968-76.
36. San Francisco Department of Public Health. HIV/AIDS Annual Report, **1997-2010**.
37. Jewell NP. Statistics for epidemiology. Boca Raton: Chapman & Hall/CRC, **2004** Texts in statistical science series).
38. R Core Team. R: A Language and Environment for Statistical Computing: R Foundation for Statistical Computing, **2012**.
39. Stevenson M, Nunes T, Sanchez J, et al. epiR: An R package for the analysis of epidemiological data, **2012**.
40. van Sighem A, Vidondo B, Glass TR, et al. Resurgence of HIV infection among men who have sex with men in Switzerland: mathematical modelling study. PLoS One **2012**; 7:e44819.
41. Bezemer D, de Wolf F, Boerlijst MC, et al. A resurgent HIV-1 epidemic among men who have sex with men in the era of potent antiretroviral therapy. AIDS **2008**; 22:1071-7.
42. McInnes CW, Druyts E, Harvard SS, et al. HIV/AIDS in Vancouver, British Columbia: a growing epidemic. Harm Reduct J **2009**; 6:5.
