# Supplementary material for: High Transmissibility During Early HIV Infection Among Men Who Have Sex With Men—San Francisco, California
Source: J Infect Dis. 2014 Dec 26;211(11):1757–60. doi: 10.1093/infdis/jiu831 (PMC4425938; doi:10.1093/infdis/jiu831)
Supplement: Supplementary Data [file supp_jiu831_jiu831supp_fig1.pdf]

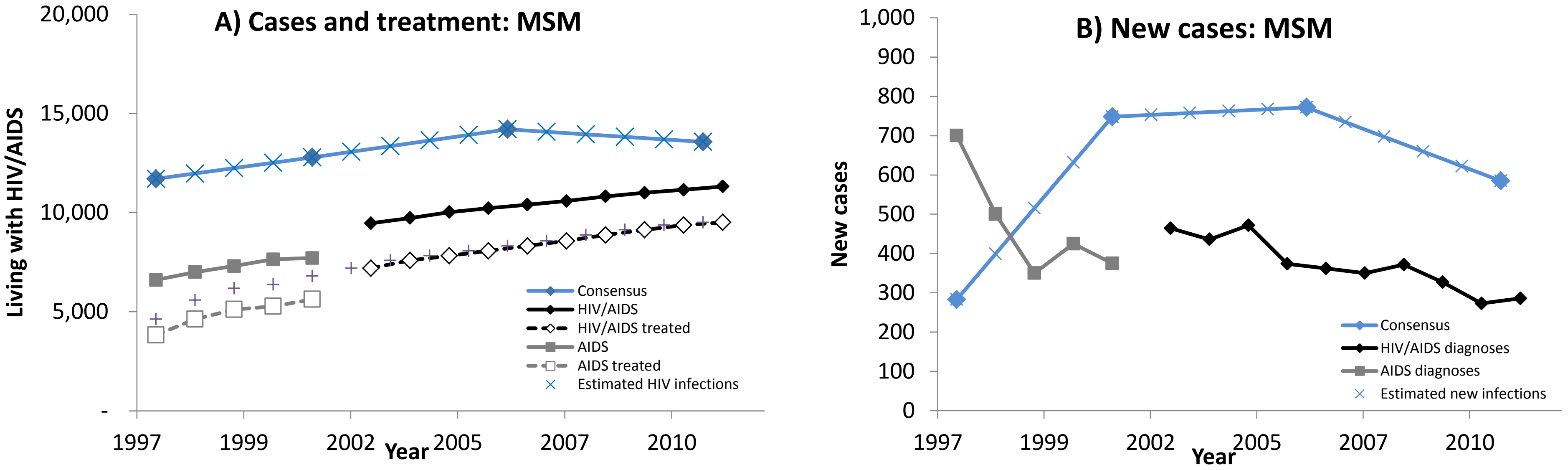

Figure S1 San Francisco Department of Public Health data on HIV infections over the period 1996 to 2010 for MSM [S36].
